# Behavioral Repertoire on a Vertical Rod—An Ethogram in *Dermacentor reticulatus* Ticks

**DOI:** 10.3390/life12122086

**Published:** 2022-12-13

**Authors:** Blažena Vargová, Natália Pipová, Miroslav Baňas, Igor Majláth, Piotr Tryjanowski, Łukasz Jankowiak, Viktória Majláthová

**Affiliations:** 1 Center for Applied Research, University Veterinary Hospital, University of Veterinary Medicine and Pharmacy in Košice, Komenského 73, 04001 Košice, Slovakia; 2 Institute of Biology and Ecology, Pavol Jozef Šafárik University in Kosice, Šrobárova 2, 04180 Kosice, Slovakia; 3 Department of Zoology, Poznan University of Life Sciences, Wojska Polskiego 71C, 60-625 Poznan, Poland; 4 Department of Vertebrate Anatomy and Zoology, University of Szczecin, Wąska 13, 71–412 Szczecin, Poland

**Keywords:** *Dermacentor reticulatus*, tick, ethogram, behavior, vertical movements

## Abstract

Ticks are important vectors of pathogens that endanger humans and animals. Study of their behavior under laboratory conditions is important for both predicting their behavior in natural conditions and understanding their involvement in transmission cycles of pathogens, which may lead to effective prevention of tick-borne disease transmission or establishment of effective preventive measures. The aim of our study was to describe the behavior of *D. reticulatus* ticks using laboratory assay. We focused on the description of individual behavioral units during their vertical movement. The assay consisted of glass beakers filled with sand and an embedded glass rod. We observed 10 different behavioral units, 4 of which have not yet been described: body posturing called “jogger”, leg grooming, and body or leg jerking. The most frequent tick behavior observed was an upwards positioning of the two front legs while the body remained motionless (88.9%). Other common observations were both horizontal (63%) and vertical (58.0%) body posturing with all legs lowered, followed by questing behavior (51.9%). Ticks spent the most time questing (75.2%), crawling (54.7%), and grooming legs on the right side (23%). We did not observe any differences between males and females.

## 1. Introduction

Ticks are obligate, blood-feeding ectoparasites parasitizing a wide spectrum of vertebrates. As vectors of pathogens, they have significant medical and veterinary importance. Many aspects of tick biology, including tick ecology, physiology, and reproduction have been studied in detail, such as that of life cycle, feeding, and searching strategy (questing, or passive waiting for hosts) [1,2,3,4,5,6,7,8].

*Dermacentor reticulatus* seems to be more thermophilic and hydrophilic than *Ixodes ricinus* while still tolerating large diurnal and seasonal temperature variation [4]. During the last few decades, the distribution of *D. reticulatus* is spreading in some regions of Europe. Remarkable spread of this species was observed in Germany, Poland, Hungary, Slovakia, Belgium, and the Netherlands [9]. Scientists considered these regions too cold to support survival and lifecycle completion in the past. Climate change may have a role in the changes in the distribution of *D. reticulatus* [10].

*Dermacentor reticulatus* is a three hosts tick. The larvae and nymph are nidicolous and the adults are exophilic. Immature ticks, compared with adults, are rarely collected by flagging. Engorged larvae molt and give rise to feeding nymphs within 30 days, with the entire life cycle being completed after just a few months [4,11]. Adult *D. reticulatus* are predominantly active from March until the summer, where their quantity on vegetation decreases, and a second wave comes in autumn from September to October. Their abundance is characterized by two peak curves in April and September [4,12,13].

Ixodid ticks spend a long time in their natural habitat and a relatively short time on hosts [1]; however, the feeding period is very import a nt for a variety of pathogens transmitted by ticks [8]. Adult *D. reticulatus* take an ambush strategy to find their hosts. Host finding is initiated by a search behavior, known as questing, in which the tick crawls an average of 55 cm [14] up vegetation to a point where it is likely to encounter a host and, then, institutes a positioning phase [15]. Ticks have sensitive chemical receptors and are attracted by host odors. Adult *Dermacentor* ticks prefer medium to large mammals and feed in clusters on the host’s skin [4]. Searching and positioning behaviors of different tick species have been studied by several authors [16,17]. It has been found that the vertical movement and location of ticks on vegetation during the initial phase of their host searching behavior is governed by a species-specific mosaic of behavioral responses to various environmental stimuli not directly related to the host. Adult ticks’ vertical movement in vegetation is controlled primarily by a combination of humidity and light [14]. Since observation of behavior in the field is complex and depends on many variables, most behavioral studies are discerned using laboratory assays, several of which were created during the last few years [18,19]. One of the most studied behaviors is locomotor activity, encompassing both exploration [18] and questing or escape behavior [20], which are impacted by physical factors, e.g., hydration [21,22], electromagnetic fields [19,23,24,25,26], or chemicals, e.g., repellents [27,28,29,30,31]. Data collected from behavioral studies conducted under laboratory conditions, which offer the possibility of variable isolation, can complement data collected from the field to avoid false correlation if behavioral or environmental measures vary with time or habitat changes [32].

The aim of our study was to describe the behavior of *D. reticulatus* ticks, using behavioral assays, under laboratory conditions. We focused on the description of individual behavioral units during their vertical movement during 180 s (s) time intervals. Vertical movement is the key to host attachment and successful parasitism. Knowledge and understanding of detailed behavior patterns is crucial for comparison in experiments where behavior can be affected by physical, chemical, or other factors.

## 2. Materials and Methods

**Figure 1:**
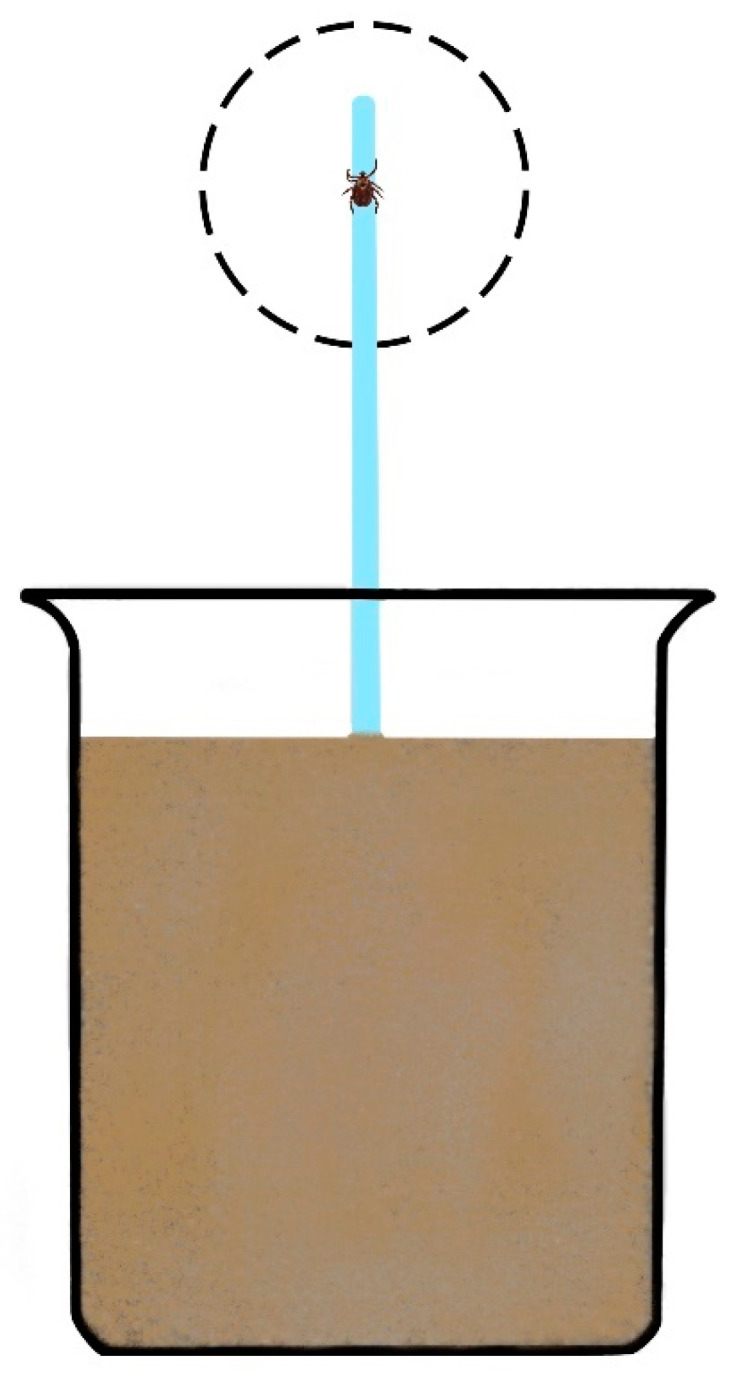
Behavioral assay. The assay consists of a glass beaker, 10 cm in diameter, filled with sand and an embedded glass rod. Animal observation was conducted in the area depicted by the dashed circle at the glass rod’s peak. The top of the rod was 20 cm above the sand. Prior to each tick placement, the rod was wiped with alcohol to eliminate pheromone cues. A humidity gradient was maintained by moistening the sand. Picture adapted with permission from Ref. [26].

We observed and described the vertical movement and detailed complex of basic behavior of the *D. reticulatus* adult ticks. Observation was performed in the behavioral assay (Figure 1) described by Vargová [26], in laboratory conditions, with a temperature of 22 °C and 60–70% humidity.

### 2.1. Animals

A total of 81 *D. reticulatus* ticks (Ixodidae, Rhipicephalinae), 36 females and 45 males, were used in the experiment. Ticks were collected by flagging with a white cotton blanket (1 m^2^) in Hrhov, Slovakia (48°36′19, 45” N; 20°45′00, 44” E) from September to November 2017. Ticks were kept in polypropylene tubes in an environmental chamber at 16 °C and 90% RH in 16:8 h light:dark regime. All ticks were maintained free of outside influences, such as odors, that could affect their behavior.

### 2.2. Experiment

For evaluation of behavior, one individual *D. reticulatus* tick was placed on the top of the glass rod in the behavioral assay. Ticks were left for 60 s to acclimatize. The behavior of ticks was video recorded by a CCD camera (model Panasonic HC-X920 Japan, Osaka, Japan) and evaluated using Solomon Coder version: beta 17.03.22. During analyses, we focused on the movement spectrum detected on the tip of the rod. In each individual tick, the movements and duration of movements were described from 180 s video sequences. Questing, turning, jerking, body positioning, grooming, crawling, leg posturing, and other exceptional behavioral manifestations were all evaluated using both a qualitative and quantitative perspective.

A total of 400 min (min) of video footage was collected, which corresponds to a total 81 ticks. Ticks were recorded without the presence of the observer to avoid the influence of external factors, which may affect tick behavior.

### 2.3. Statistical Analyses

We compared movement occurrences between females and males by using a generalized linear model with binomial error distribution and duration of movement by using a general linear model with normal error distribution. As a significant threshold, *p* < 0.05 was taken.

## 3. Results

### 3.1. Behavioural Repertoire

We observed 10 behavioral units of either the whole body or just parts of the body. All observed movements of *D. reticulatus* ticks on the vertical glass rod are described in detail in Table 1.

### 3.2. Movement Occurrence and Duration

The occurrence of individual behaviors or positions and duration of movements were evaluated (Table 2).

The most frequent behaviors in *D. reticulatus* ticks were “first pair of legs posture: up” (88.9%), “tick body position: “horizontal” (63%), vertical” (58.0%), and “questing” (51.9%).

For the first time, we observed grooming behavior in ticks. They preferred the cleaning of the first pair of legs. Grooming behavior was observed in 30 out of 81 ticks, and the mean time of grooming was 41 s.

The behaviors that were recorded the least were newly observed movements, such as a body posture called “jogger” (2.5%) and body/leg jerking (3.7%).

From the perspective of behavior duration, ticks spent the most time questing (75.2%), crawling (54.7%), and grooming of legs (1 + 2) on the right side (23%).

We did not observe any differences in behavioral manifestations between males and females; however, for the first pair of leg postures, up and crawling, we noted slightly higher movement occurrence in females.

## 4. Discussion

Even though their behavior is not well known, the *D. reticulatus* ticks in this study showed a considerably rich set of behaviors relating to vertical movement on the stick and remaining in the top position, which are essential for finding a host. We observed 10 different behavioral units, 4 of which have not yet been described and are likely unrelated to locating a host. The purpose of some behaviors was derived from similar behavior observed and described in other animal taxa, e.g., grooming of legs in fruit flies [33].

Classical ethogram studies describe known behaviors such as interaction behavior between different species, reproductive and sexual behavior, food-catching behavior, and many other behavior types of different species from bacteria, invertebrates, and vertebrates [34,35,36]. Several apparatuses, arenas, or chambers were designed and are in use to study the behavior of invertebrates [37,38]. Some of them are focused on the reactions from the olfactory stimuli, such as Y and T mazes or multichoice olfactometers, which measure choices performed by invertebrates in response to attractants [31,39]. Currently, automatized tools are important innovative implements which store digital data and enable new insight into behavior [33,40]. Despite highly sophisticated mazes and software-based tools becoming a part of ethological research, technically simple constructions can still greatly aid in the observation of basic behavioral manifestations.

The importance of *D. reticulatus* ticks for veterinary and human medicine is significant, as *D. reticulatus* is a vector of variety of pathogens including viruses, bacteria, and parasites [4]. *Dermacentor reticulatus* ticks attach and feed on a wide range of hosts, including wild mammals, domesticated mammals, and in some cases, humans [4,28]. Thus, it is also important to understand behavioral patterns in tick species to prevent the spread of disease.

Additionally, it is crucial to know the precise behavioral pattern of ticks for planning behavioral experiments in order to predict and evaluate changes caused by factors under laboratory conditions. We apply our observations as the basis for other experiments.

The most important part of a tick’s behavior is climbing, questing, and posturing of the first pair of legs [41]. Questing of ticks involves leaving the ground microhabitat and climbing up on vegetation, where it adopts a sit and wait tactic for finding a host and survival. Understanding the ecology and evolution of questing is key to discerning the fluctuations in risk of tick-borne diseases [40].

According to our observations, there was no difference in the behavioral pattern between genders in the vertical position. The primary questing task of ticks is to maximize contact with the host for several needs such as taking a blood meal and finding a partner for reproduction [42].

## 5. Conclusions

The behavioral complex of *D. reticulatus* ticks has not yet been described. We were the first to describe the specific behavior of ticks during vertical movement. In this paper, we observed 10 types of behavior and postures. All recorded tick behaviors and postures were described in detail. We found four movements that, although repeated infrequently, were rather extraordinary: body posturing called “jogger”, leg cleaning, and body or leg jerking. We can’t yet elucidate what these movements mean and what significance they have for the ticks; however, further research is needed to better understand tick behavior. This understanding may promote knowledge of the overall ecology of organisms in nature.

## Figures and Tables

**Figure 2:**
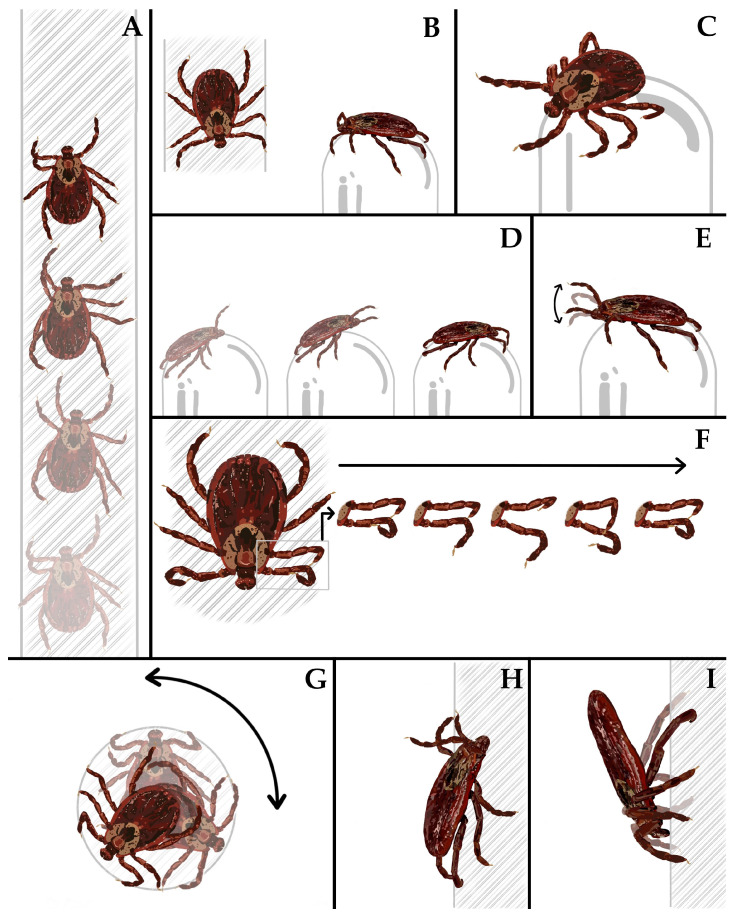
Movements and positions of D. reticulatus ticks observed during the experiment: (**A**)—Tick body position; (**B**)—Questing; (**C**)—Turning clockwise/counterclockwise; (**D**)—Body jerking; (**E**)—Leg jerking; (**F**)—Grooming; (**G**)—Crawling; (**H**)—First pair of legs posture; (**I**)—Jogger. For a detailed description of the movements and postures, see Table 1.

**Table 1:** List of the behavioral repertoire on the vertical rod of *D. reticulatus* ticks. R-right side of the body, L-left side of the body.

Behavior	Description
Crawling Tick body position Questing Body jerking	A slow walking upwards or downwards on the rod (Figure 2A). The position of the tick on the top of the rod, either vertical or horizontal (Figure 2B). The first pair of the tick’s legs are lifted in the environment and slightly moving to trap the stimuli, the other 3 pairs of legs are firmly attached on the rod (Figure 2C). Sudden jerky motion of the body, occurred after a few seconds of freezing in a vertical position (Figure 2D).
Legs jerking Grooming Turning	Sudden jerky motion of the first pair of legs, occurred after a few seconds of freezing in a vertical or horizontal position (Figure 2E). Leg cleaning movements of different pairs of legs and on different sides of the body (Figure 2F) in the combinations: 1L + 2L along with 1R + 2R; 3L + 4L along with 3R + 4R; 3L + 4L; 3R + 4R. The tick rotates around its axis on one place in a horizontal or vertical position (Figure 2G). This movement is performed with all legs down, on the stick, and leg pairs are moved alternately.
Turning clockwise Turning counterclockwise First pair of legs posture Jogger	This movement is a part of the turning movement and occurs in the clockwise direction (Figure 2G). This movement is a part of the turning movement and occurs in the counterclockwise direction (Figure 2G). Up or down position of the first pair of legs, not questing. Legs are withdrawn or half open (Figure 2H). The tick is in the vertical position, the *basis capituli* is in downward position, and the whole body is stretched to a semicircle or letter “S”. All legs are on the stick, *basis capituli* with hypostome presses down and the area of the festoons of tick pushes upwards (Figure 2I). Movement observed rarely.

**Table 2:** Ethogram showing different types of observed behavior. Occ—frequency of occurrence of a given behavior; dur—duration: the sum of time across all individuals manifesting specific behavior and the percentage of duration of the observed specific behavior out of the total duration of the experiment in the individuals which performed the behavior; durM—mean duration of behavior in ticks manifesting specific behavior; num—total number of specific movement. Observation period per individual was 180 s.

		Female		Male		All Individuals		F vs. M
Type of Behavior		%	Sum/M	%	Sum/M	%	Sum/M	*p*
Questing	occ	61.1	22	44.4	20	51.9	42	0.138
	dur	74.2	2938 s	76.3	2748 s	75.2	5686 s	0.235
	durM		133.5 s		137.4 s		135.4 s	
Turning clockwise	occ	19.4	7	8.9	4	13.6	11	0.178
Turning counter clockwise	occ	13.9	5	6.7	3	9.9	8	0.289
Body jerking	occ	8.3	3	0.0	0	3.7	3	
Legs jerking	occ	5.6	2	2.2	1	3.7	3	
Vertical body position	occ	63.9	23	53.3	24	58.0	47	0.340
Horizontal body position	occ	58.3	21	66.7	30	63.0	51	0.441
Grooming: Legs 1 + 2	occ	27.8	10	35.6	16	32.1	26	0.509
	dur	23	410 s	23	662 s	23	1072 s	0.608
	dur*M*		41 s		41 s		41 s	
Legs 3 + 4 left	occ	2.8	1	2.2	1	2.5	2	
	dur	0.7/24.4	44 s	0.4/16.7	30 s	0.5/20.6	74 s	
	dur*M*		44 s		30 s		37 s	
Legs 3 + 4 left	occ	0.0	0	2.2	1	1.2	1	
	dur	0.0	0 s	0.6/25.6	46 s	0.3/25.6	46 s	
	dur*M*		0 s		46 s		46 s	
Legs 3 + 4	occ	2.8	1	0.0	0	1.2	1	
	dur	0.5/16.7	30 s	0.0	0 s	0.2/16.7	30 s	
	dur *M*		30 s		0 s		30 s	
Crawling	occ	41.7	15	22.2	10	30.9	25	0.063
	dur	46.5	1255 s	67	1206 s	54.7	2461 s	0.537
	dur*M*		84 s		121 s		98 s	
First pair of legs posture: up	occ	97.2	35	82.2	37	88.9	72	0.062
down	occ	0.0	0	17.8	8	9.9	8	
Jogger	occ	2.8	1	2.2	1	2.5	2	
	num		8		3		11	
Total individuals			36		45		81	
Total observation duration [s]			6480		8100		14,580	

## Data Availability

Not applicable.
